# All-in-one properties of an anticancer-covered airway stent for the prevention of malignant central airway obstruction

**DOI:** 10.1063/5.0157341

**Published:** 2023-09-13

**Authors:** Zhaonan Li, Wenguang Zhang, Dechao Jiao, Chuan Tian, Kaihao Xu, Haidong Zhu, Xinwei Han

**Affiliations:** 1 Center of Interventional Radiology and Vascular Surgery, Department of Radiology, Zhongda Hospital, Medical School, Southeast University, Nanjing 210009, China; 2 Department of Interventional Radiology, First Affiliated Hospital of Zhengzhou University, Zhengzhou 450001, China

## Abstract

Malignant central airway obstruction (MCAO) resulting from tumor metastasis and compression severely impairs respiration, posing life-threatening risks. To address this, we employed a synergistic modification strategy, combining cisplatin (CIS) and silver nanoparticles (AgNPs). Polycaprolactone (PCL) served as a drug carrier, enabling the preparation of a functional CIS@AgNPs@PCL fiber membrane-covered airway stent via electrospinning. This approach aimed to enhance the patency rate of MCAO. Characterization via ATR-FTIR, scanning electron microscope-energy-dispersive spectroscopy, and transmission electron microscope confirmed successful immobilization of CIS and AgNPs onto the stent surface. CIS@AgNPs@PCL substantially suppressed non-small cell lung cancer cells (A549), causing DNA damage, ultrastructural disruption, and over 50% apoptosis in 48 h. It also displayed potent antibacterial activity against *Staphylococcus aureus*, *Pseudomonas aeruginosa*, and *Candida albicans* biofilms. A mouse subcutaneous tumor recurrence model assessed anti-cancer efficacy. CIS@AgNPs@PCL fiber-covered stents significantly inhibited lung cancer tissue and enhanced anti-cancer effects by up-regulating caspase-3 and Bax, while down-regulating Bcl-2. This study's functional airway stent provides a proof-of-concept for an integrated anti-cancer and antibacterial strategy. It promptly restores the lumen, inhibits biofilm formation, prevents tumor progression, and improves postoperative MCAO patency.

## I. INTRODUCTION

Malignant central airway obstruction (MCAO) poses a challenging issue for medical and surgical specialists who treat thoracic diseases.^1^ Patients with MCAO often suffer from dyspnea, which can be life-threatening in severe cases.^2^ The prevalence of lung cancer (LC) has contributed to the rising incidence of this disorder. Unfortunately, malignancies that cause main bronchial obstruction are often not amenable to surgery, and localized ablation therapy, such as cryoablation, electrocautery, and laser therapy, provides limited treatment options with uncertain long-term efficacy.^3–5^ For elderly patients or those with poor cardiopulmonary conditions who cannot undergo ablation therapy, airway stenting may be the most effective option to quickly relieve airflow limitation symptoms and reinforce the tube wall's support force. However, stenting as a palliative means of relieving symptoms is not equipped to treat intratracheal tumor invasion or metastasis, which poses a challenging problem for clinicians.^6^ Therefore, developing a functional airway-covered stent with anticancer properties could be a reliable solution to address the aforementioned problems.

In general, a significant proportion of LC patients develop MCAO symptoms during the disease's course, and up to 40% of LC-related deaths are due to local disease progression.^7,8^ Cisplatin (CIS)-based chemotherapy is currently the most widely used regimen in treating advanced LC, resulting in significantly improved median overall survival (OS) compared to other supportive therapies.^9,10^ CIS is also the standard of care for most patients with resected stage II or III LC. According to the International Adjuvant Lung Cancer Trial (IALT), using CIS-based adjuvant chemotherapy after surgical resection of LC can increase the 5-year survival rate of patients by 4% and the disease-free survival rate by 5%.^11–13^ However, conventional systemic chemotherapy using CIS has been unsatisfactory in rapidly restoring airway patency with MCAO. Considering the aforementioned findings and the challenge of increased tumor drug resistance with intravenous chemotherapy alone, combining CIS with silver nanoparticles (AgNPs) may be a reliable approach to improve treatment efficiency.

The application of AgNPs in airway stents offers dual benefits. First, AgNPs possess anti-cancer properties and can serve as adjuvant chemotherapy agents, enhancing tumor-killing effects and chemosensitivity.^14,15^ AgNPs induce morphological alterations in cells by reducing cellular metabolism, increasing oxidative stress, and ultimately leading to DNA damage and apoptosis.^16^ For instance, El-Naggar *et al.* demonstrated that intraperitoneal injection of biosynthetic AgNPs significantly reduced malignant cells and improved the survival rate of Dalton's lymphoma-bearing mice with ascites.^17^ Sriram *et al.* showed that intravenous administration of nontoxic doses of AgNPs resulted in a significant reduction in tumor proliferation in mice with triple-negative breast cancer.^18^ Second, AgNPs are commonly used in stent graft applications due to their potent antibacterial properties. Prolonged stent implantation in the airway can lead to bacterial biofilm formation and subsequent stent-associated infection. Biofilms containing lipids can block the penetration of water-soluble antibiotics, rendering commonly used antibiotic therapy ineffective against biofilm-related infections.^19^ Therefore, research indicates that stents containing AgNPs can effectively prevent bacterial adhesion and subsequent biofilm formation.^20–22^

In this study, a functional fiber membrane-covered airway stent (CIS@AgNPs@PCL) with multiple anticancer and antibacterial properties was constructed using electrospinning. Interdisciplinary techniques were employed to comprehensively investigate the physicochemical and biological activities of the fiber membranes. All stents loaded with CIS and AgNPs exhibited satisfactory tumor cell inhibition rates *in vitro*, while the AgNP-containing fibrous membranes remarkably suppressed the formation of bacterial biofilms. Airway tumor recurrence and microbial colonization of the stent are major factors affecting patient prognosis. However, the current research on the anticancer/antibacterial effects of airway stents still remains limited. The CIS@AgNPs@PCL stent constructed in this study demonstrates multiple anticancer properties with sustained and gradual drug release, aiming to achieve tumor suppression and improve the therapeutic efficacy of MCAO.

## II. RESULTS AND DISCUSSION

### A. Characterization of electrospun fiber membrane

**FIG. 1.**
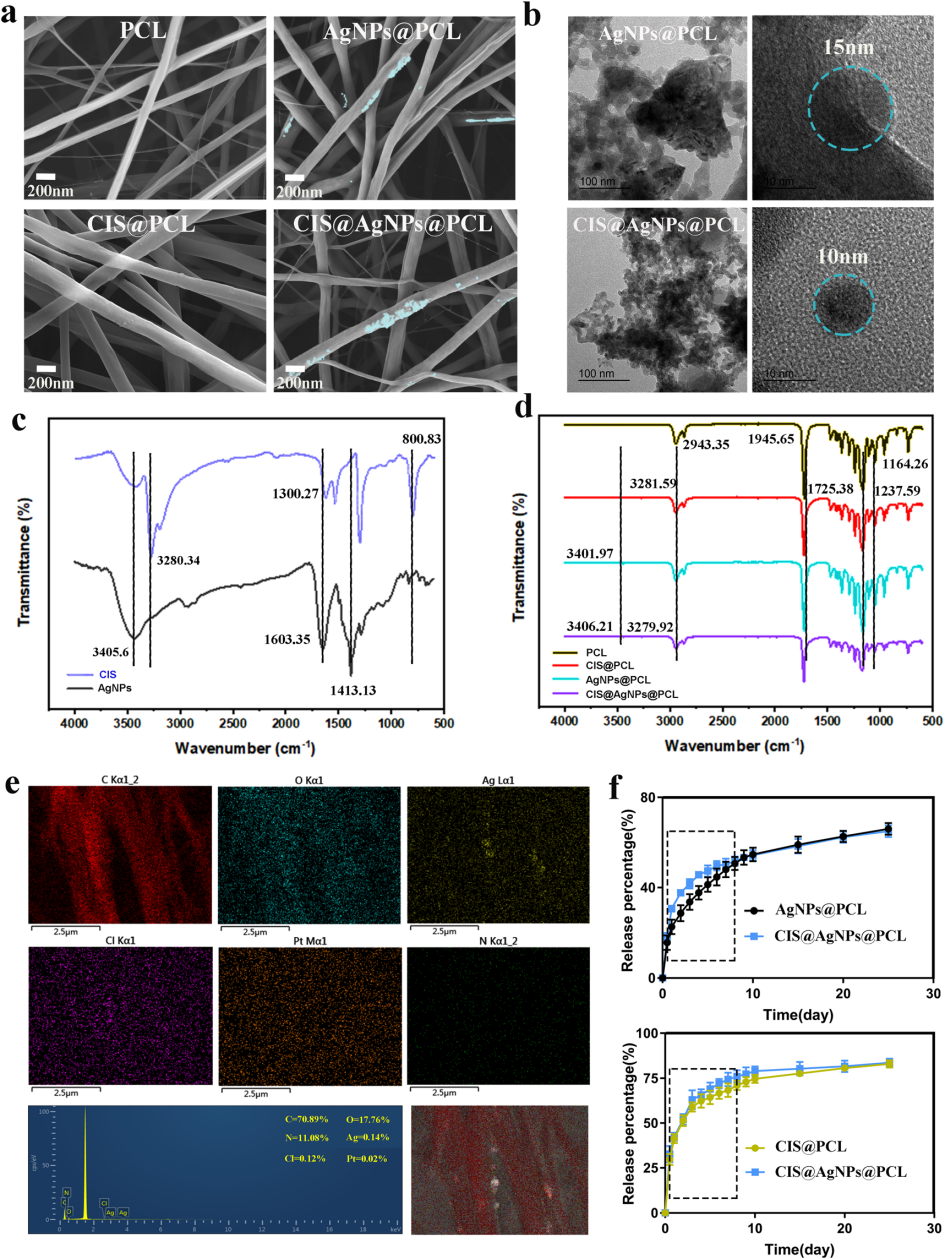
Characterization of four sets of electrospun fiber-covered airway stents. (a) The four groups of membranes were observed under the scanning electron microscope (SEM), the fiber filaments were smooth and flat, and AgNPs were stably wrapped in the fiber filaments. (b) The diameter of AgNPs in AgNPs@PCL and CIS@AgNPs@PCL in the stent capsule was observed by a transmission electron microscope (TEM). FTIR (c) and (d) spectra of AgNPs powder, CIS powder, and the membrane of PCL, AgNPs@PCL, CIS@PCL, and CIS@AgNPs@PCL, respectively. EDS spectrum (e) and elemental mapping (f). Drug release of CIS and AgNPs from different drug-loaded fiber membranes.

Using electrospinning technology, PCL-based drug delivery stent systems were constructed, incorporating PCL, AgNPs@PCL, CIS@PCL, and CIS@AgNPs@PCL as membrane-coated airway stents. The morphological structures of the membranes were investigated using SEM. As shown in Fig. 1(a), all fiber bundle samples demonstrated a randomly interconnected structure with uniform fiber size. The diameters of PCL, AgNPs@PCL, CIS@PCL, and CIS@AgNPs@PCL fiber bundles were, on average, 273.2 ± 110.2, 246.8 ± 96.7, 212.3 ± 104.5, and 289.7 ± 101.7 nm, respectively. On the AgNPs@PCL and CIS@AgNPs@PCL samples, the AgNPs were encapsulated by fiber bundles. The TEM images of Fig. 1(b) demonstrate that the AgNPs in AgNPs@PCL and CIS@AgNPs@PCL were encapsulated by fiber bundles with sizes ranging from 15 to 20 nm, showing that the AgNPs in this process had good properties. Furthermore, the TEM images indicated that the AgNPs had a spherical morphology, which was in agreement with previously published reports.^23^ Studies have found that the spherical structure of AgNPs can better reflect the strong antibacterial properties of AgNPs than other morphologies (linear, triangular, and cubic).^24,25^ This phenomenon may be related to the larger effective contact area and better reactive surface of the spherical structure. Moreover, it has been shown that the anticancer and antibacterial properties of AgNPs are impacted by their intrinsic properties, such as morphology, size, and surface charge, with smaller particle diameter linked to higher bioactive mass.^26^

**FIG. 2.**
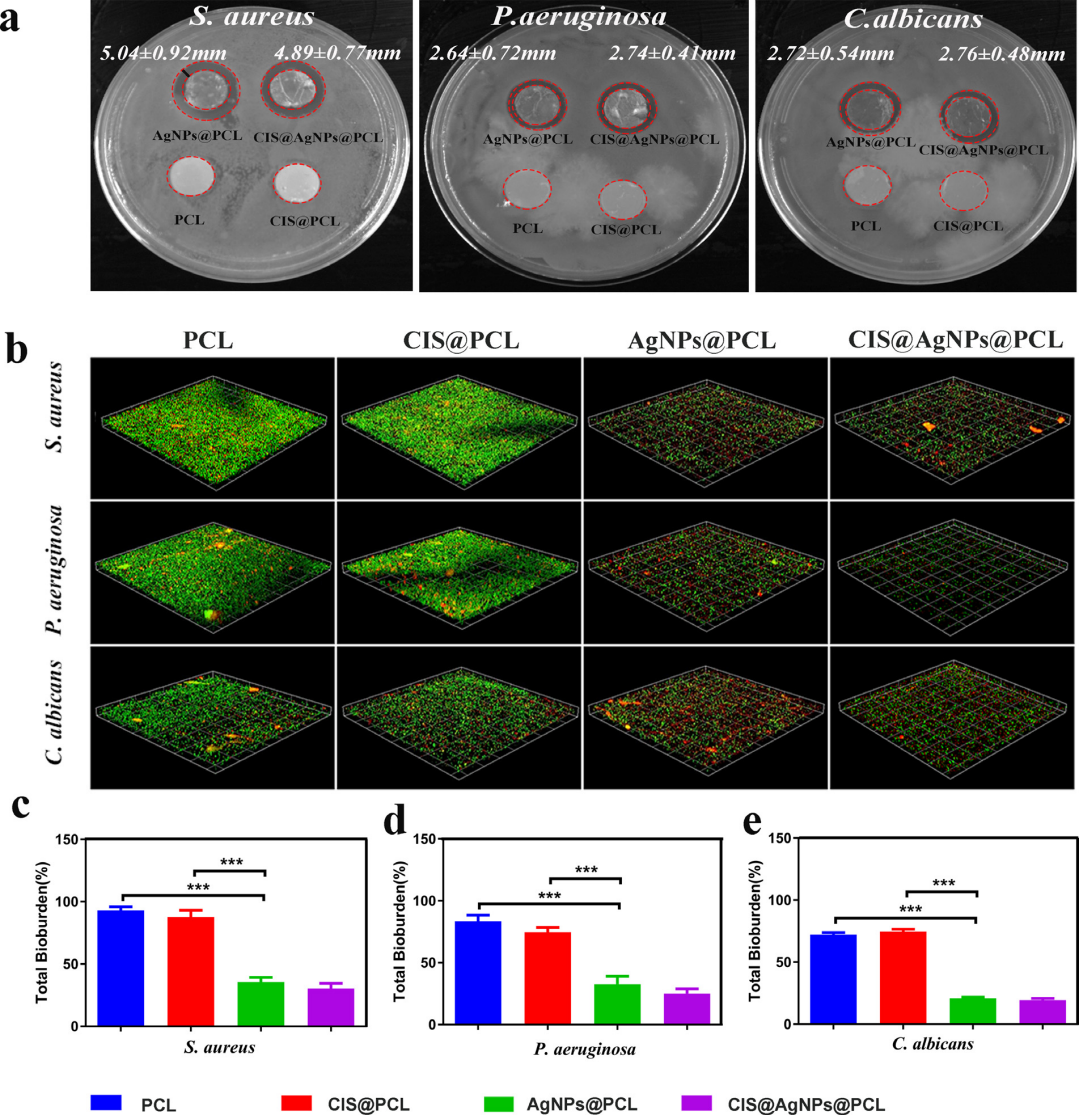
Antibacterial properties of fiber membranes. (a) Experimental results of the inhibition zones of four groups of fiber membranes with *S. aureus*, *P. aeruginosa*, and *C. albicans*. (b) Laser confocal microscopy was used to analyze the total microbial load of four groups of fiber membranes incubated with *S. aureus, P. aeruginosa*, and *C. albicans*, respectively. (c)–(e) Statistical analysis of the total microbial load on the four groups of membranes; *, P < 0.05; **, P < 0.005; and ***, P < 0.0005.

As depicted in Fig. 1(e), the EDS element distribution of the constructed CIS@AgNPs@PCL film contained C, N, O, Ag, Cl, and Pt, demonstrating that CIS and AgNPs were effectively embedded in the fiber structure and remained stable after electrospinning. In addition, the FTIR spectrum of CIS indicated the characteristic absorption peaks of N–H stretching vibration at 3281.35, 1301.27, and 802.83 cm^−1^, while AgNPs exhibited the absorption peaks at 3405.6, 1602.35, and 1413.13 cm^−1^ showed a prominent peak [Fig. 1(c)]. Moreover, the fiber membranes AgNPs@PCL, CIS@PCL, and CIS@AgNPs@PCL all displayed the characteristic absorption peaks of PCL, AgNPs, and CIS [Fig. 1(d)], suggesting that AgNPs and CIS were successfully incorporated into the fiber membrane, which fulfilled the requirements of subsequent experiments.

The drug release experiment is an important part of this study. The drug release of PCL, AgNPs@PCL, CIS@PCL, and CIS@AgNPs@PCL was measured in a constant temperature PBS solution with a pH of 7.4. As shown in Fig. 1(f), the release behavior of CIS and AgNPs in the fibrous membrane AgNPs@PCL, CIS@PCL, and CIS@AgNPs@PCL is mainly divided into two sections, namely, the burst release section and the sustained release section. Specifically, the proportions of AgNPs released by AgNPs@PCL and CIS@AgNPs@PCL in the first eight days were 51.2% ± 3.3% and 50.8% ± 2.6%, respectively. In addition, the CIS release of CIS@PCL and CIS@AgNPs@PCL in the first eight days was 70.5% ± 3.2% and 75.8% ± 2.4%, respectively. For the rest of the time thereafter, the drug continued to be released slowly from the fiber membrane. In fact, because the airway tissue is in a closed and narrow environment, the stents implanted in the airway do not have strong liquid flushing, so the released drugs will persist and accumulate on the airway surface, maintaining a higher concentration of drug efficacy to achieve the purpose of local treatment.

### B. Evaluation of the *in vitro* antimicrobial effects

**FIG. 3.**
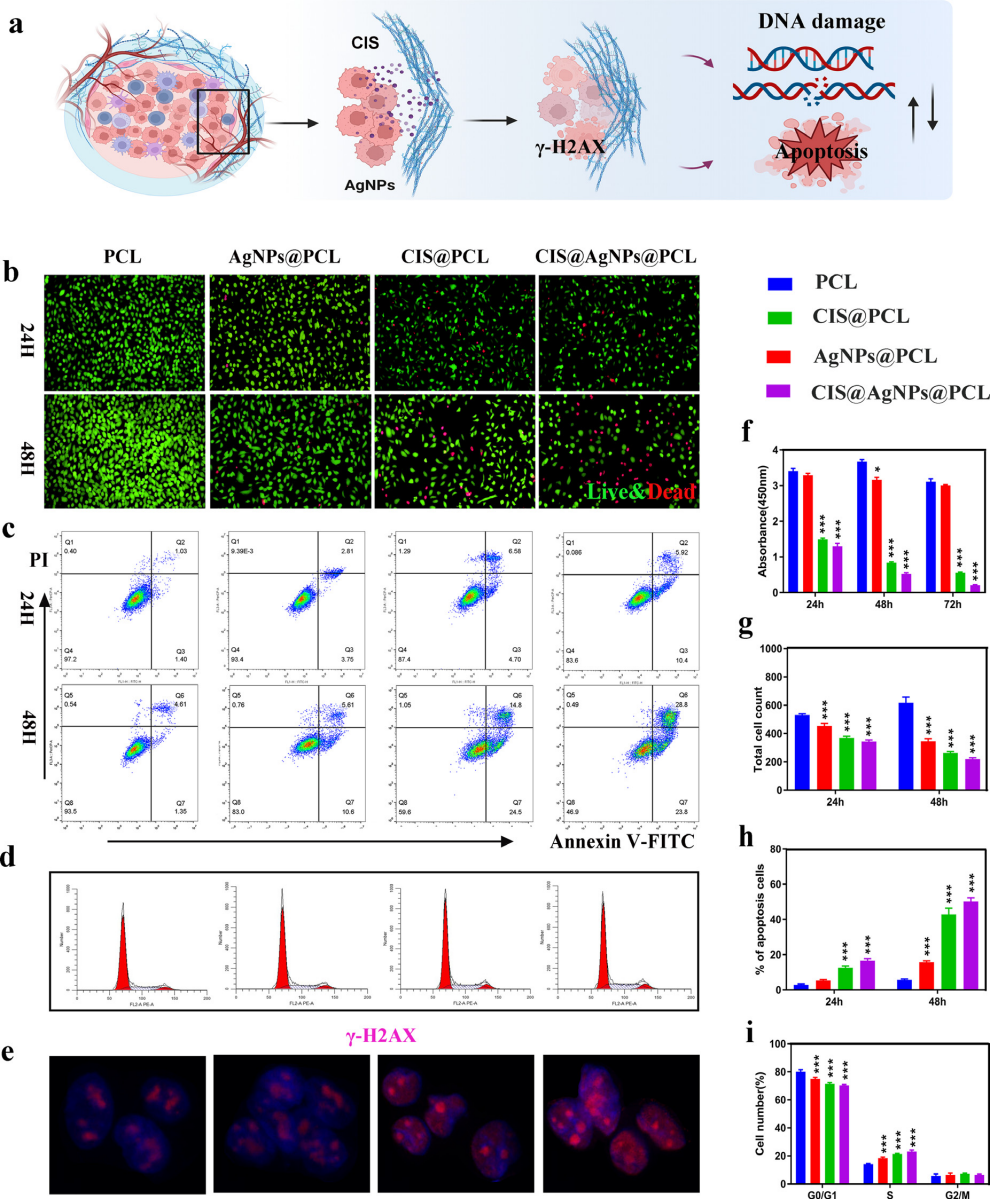
*In vitro* antitumor experiments. (a) Antitumor mechanism diagram of AgNPs cooperating with CIS. (b) Live/dead staining results and statistical analysis (g) of A549 cells cultured with PCL, AgNPs@PCL, CIS@PCL, and CIS@AgNPs@PCL sustained-release solution for 24 and 48 h, respectively. (c) The effects of PCL, AgNPs@PCL, CIS@PCL, and CIS@AgNPs@PCL sustained-release solution on the apoptosis state of A549 cells were detected by flow cytometry and statistical analysis (h). (d) Cell cycle test results and related statistical analysis of four groups of material release solution on A549 (i). (f) CCK-8 assay of A549 cells at 1, 2, and 3 d, respectively. (e) Expression of γ-H2AX immunofluorescence in A549 cells by PCL, AgNPs@PCL, CIS@PCL, and CIS@AgNPs@PCL sustained-release solution. ^*^p < 0.05, ^**^p < 0.005, and ^***^p < 0.0005.

The issue of stent-associated infection following airway stenting cannot be effectively resolved, primarily due to the colonization of bacterial biofilm on the stent's surface. In a molecular analysis of airway stent biofilms, it was observed that the prolonged symbiotic interaction between stents and microorganisms promotes the rapid formation of bacterial biofilm.^27^ Several studies suggest that bacterial biofilms impede the penetration of water-soluble antibiotics, rendering them less effective against pathogens. Moreover, biofilms exhibit increased resistance to host immune responses and antimicrobial agents compared to other bacterial forms, resulting in reduced efficacy of standard antibiotic therapy.^28,29^ Therefore, by incorporating potent antimicrobial substances onto the surface of the coated airway stent, the formation of bacterial biofilm can be delayed or prevented, leading to a decrease in stent-related infection rates. In this study, silver nanoparticles (AgNPs) were utilized to confer the airway stent with broad-spectrum antibacterial properties. *In vitro* antibacterial experiments were conducted to evaluate the effectiveness against common respiratory pathogens, such as *P. aeruginosa*, S. aureus, and *C. albicans*, as depicted in Fig. 3(a).

The antimicrobial properties of the fiber membranes were studied by observing the change in the diameter of the antimicrobial zone. *P. aeruginosa*, *S. aureus*, and *C. albicans* were grown on the agar plates around the membranes. There were no antimicrobial zones around the PCL and CIS@PCL, and the three types of microorganisms proliferated and covered the entire Petri dish. In contrast, obvious antimicrobial zones appeared around the silver-loaded fiber membranes (AgNPs@PCL and CIS@AgNPs@PCL), suggesting the excellent antimicrobial performance of the AgNP-containing fiber membranes in a solid Petri dish.

The live/dead viability assay results of bacterial biofilms were determined by COMSTAT software analysis. A merged image of live/dead microbial biofilm staining is shown in Fig. 3(b). In the PCL group, *P. aeruginosa*, *S. aureus*, and *C. albicans* were mostly stained green and displayed the strongest fluorescence intensity, and the total microbial load on the surface of the fiber membrane was the highest. With the addition of AgNPs, live microorganisms gradually weakened, while the red fluorescence indicating dead microorganisms enhanced relative to that observed on the PCL membrane. Specifically, compared to PCL and CIS@PCL, the presence of AgNPs in AgNPs@PCL resulted in an obvious reduction in the total microbial load (P < 0.0005). However, there was no major difference between the total microbial load in CIS@AgNPs@PCL and AgNPs@PCL (P > 0.05). The incubation of PCL, CIS@PCL, AgNPs@PCL, and CIS@AgNPs@PCL with *S. aureus* for 72 h showed that the proportions of microbial load were as follows: 92.950% ± 2.625%, 87.725% ± 4.739%, 35.525% ± 3.333%, and 30.425% ± 3.581%, respectively [Fig. 3(c)]. Following a 72-h incubation with *P. aeruginosa*, the proportions of microbial load on PCL, CIS@PCL, AgNPs@PCL, and CIS@AgNPs@PCL were 83.400% ± 4.317%, 74.675% ± 3.296%, 32.725% ± 5.442%, and 25.100% ± 3.225%, respectively [Fig. 3(d)]. Similarly, after incubating with *C. albicans* for 72 h, the proportions of microbial load on PCL, CIS@PCL, AgNPs@PCL, and CIS@AgNPs@PCL were 72.050% ± 3.024%, 74.675% ± 3.296%, 20.775% ± 1.895%, and 19.475% ± 2.354%, respectively [Fig. 3(e)]. Bacterial biofilms hinder the efficacy of chemotherapy drugs on tumors and contribute to a remarkable increase in the bacterial/tumor resistance. The synergistic interaction between CIS and AgNPs will substantially mitigate this adverse reaction by inhibiting biofilm formation and enhancing the anti-cancer effect. Quantitative statistical analysis demonstrates that the stent covered with the AgNPs-containing fiber membrane exhibits significant efficacy in inhibiting biofilm formation by common pathogenic bacteria in the airway such as *P. aeruginosa*, *S. aureus*, and *C. albicans*. This suggests that CIS@AgNPs@PCL possesses the capability to prevent microbial colonization following stent placement and strengthen the therapeutic effect on tumors.

### C. *In vitro* antitumor experiments

**FIG. 4.**
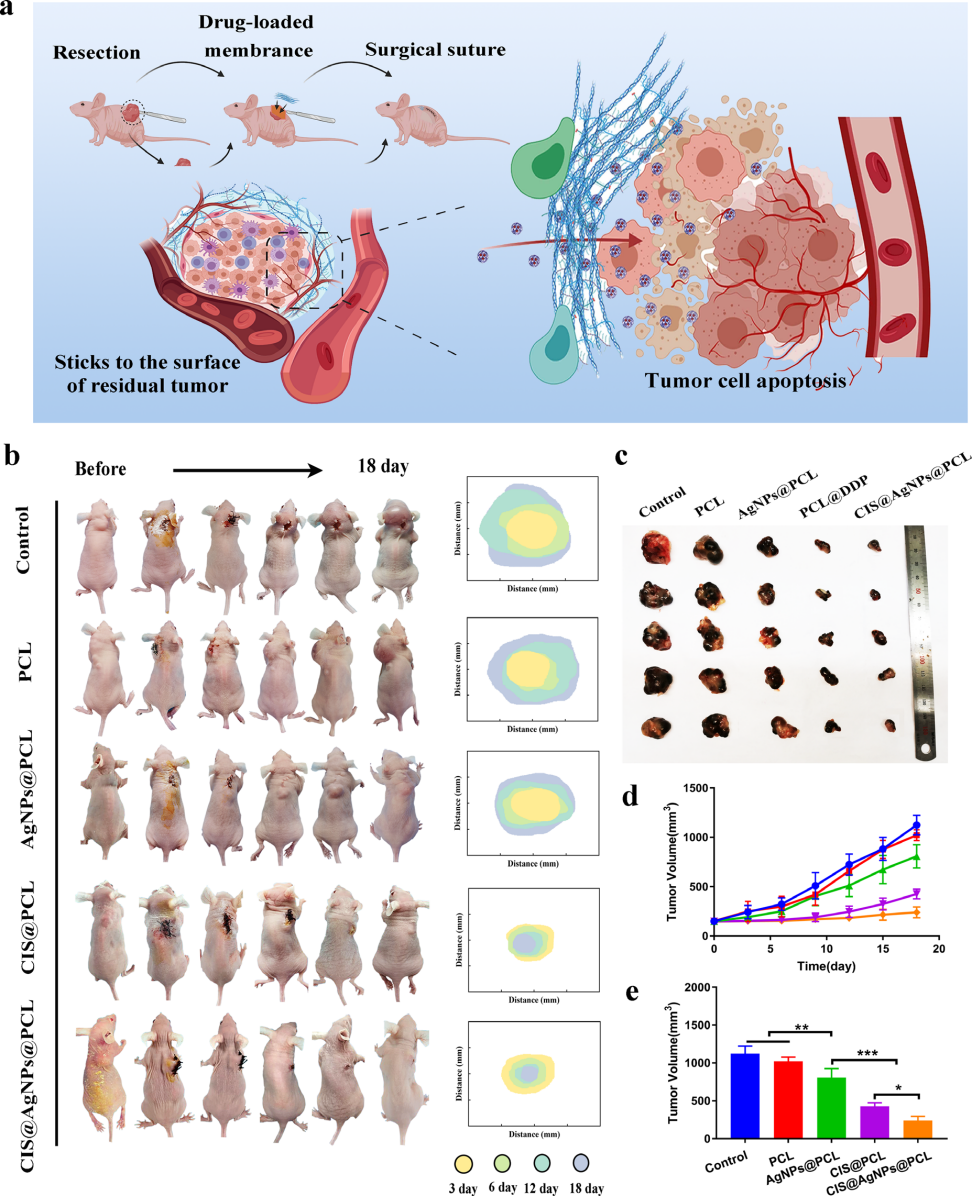
*In vivo* antitumor efficacy of different fiber membranes. (a) Schematic diagram of sustained drug release therapy of CIS combined with AgNPs synergistically modified fiber membrane CIS@AgNPs@PCL attached to the resected tumor surface. (b) Typical mice of LC recurrence model and tumor volume change diagram. (c) *In vitro* tumor photos of five groups of experimental mice. (d) Changes in tumor volumes of subcutaneous xenografts in nude mice treated with PCL, AgNPs@PCL, CIS@PCL, and CIS@AgNPs@PCL or nothing as the control. (e) Statistical analysis of comparison of tumor volumes on the day of the sacrifice of five groups of tumor-bearing nude mice; ^*^, P < 0.05; ^**^, P < 0.005; and ^***^, P < 0.0005.

The study aims to not only highlight the remarkable antimicrobial properties of airway stents but also to address the crucial issue of preventing the reemergence of intratracheal tumors. Malignant CAO caused by lung cancer recurrence or metastasis poses a significant challenge as the current treatment options are limited.^30^ To evaluate the stents' anticancer efficacy, A549 cells were employed as model cells, and their response to PCL, AgNPs@PCL, CIS@PCL, and CIS@AgNPs@PCL release buffer was observed. This evaluation will aid in determining the stents' potential as a viable treatment option for the prevention of LC tumor recurrence [Fig. 4(a)].

To assess the impact of culture in a release buffer on A549 cell apoptosis, an apoptosis staining kit was employed for 24-h and 48-h intervals. The findings indicated that all fiber membranes, excluding PCL, stimulated the apoptosis of A549 cells. Moreover, the number of apoptotic cells increased progressively with longer incubation periods [Fig. 4(c)]. In detail, after 24 h of incubation, the percentages of apoptotic cells in PCL, AgNPs@PCL, CIS@PCL, and CIS@AgNPs@PCL were 2.93% ± 0.39%, 5.34% ± 0.48%, 12.53% ± 0.10%, and 16.57% ± 1.03%, respectively. After 48 h of incubation, the percentages of apoptotic cells in PCL, AgNPs@PCL, CIS@PCL, and CIS@AgNPs@PCL were 5.67% ± 0.61%, 15.66% ± 0.71%, 42.81% ± 0.72%, and 50.16% ± 1.85%, respectively [Fig. 4(h)]. Apoptosis is the result of tightly regulated cellular processes, characterized by cell membrane blebbing, cell shrinkage, and DNA fragmentation. The aforementioned results confirm that both AgNPs and CIS released into the buffer solution can induce apoptosis in A549 cells. Importantly, the sustained-release solution of CIS@AgNPs@PCL exhibits the most pronounced tumor cell apoptosis throughout the experimental period. The precise signaling pathways and molecular mechanisms underlying cell apoptosis require further exploration to ascertain the exact cellular processes involved.

As shown in Fig. 4(b), the killing ability of different fiber membranes on tumor cells was evaluated by using the cell viability/dead staining kit. The highest number of dead cells with red fluorescence, as revealed by fluorescence microscope, was CIS@AgNPs@PCL. In detail, with AgNPs and CIS being added to the fiber membrane, the number of live cells in AgNPs@PCL and CIS@PCL gradually reduced. Moreover, due to the possible synergistic anti-cancer properties of AgNPs and CIS, the number of live cells with green fluorescence in CIS@AgNPs@PCL decreased most significantly [Fig. 4(g)].

In addition, the CCK-8 assay was provided to analyze the proliferation changes of the fiber membrane in lung cancer A549 cells. Figure 4(f) showed that A549 incubated with membrane release buffer for 24, 48, and 72 h displayed an obvious time-dependent decline in the cell OD value, that is, the cell viability decreased with the extension of co-incubation time. Specifically, the OD value of AgNPs@PCL was slightly lower than that of the PCL group, indicating that AgNPs had a certain inhibitory effect on A549 cells. Moreover, the OD value of CIS@PCL and CIS@AgNPs@PCL was seriously reduced, indicating that CIS released in the buffer was the main substance to inhibit A549 proliferation, and CIS@AgNPs@PCL with synergistic effect could show the best antiproliferative effect on A549. With the long-term release of CIS and AgNPs, the activity of tumor cells was obviously inhibited.

AgNPs have been demonstrated to induce cell cycle arrest with some studies reporting S-phase arrest while others indicating G2-phase arrest, suggesting that the impact on the cell cycle may depend on the cell type. In this study, tumor cells treated with fiber membranes containing AgNPs and CIS exhibited varying degrees of cell cycle arrest among the different groups. Specifically, compared to the PCL group, the AgNPs@PCL group showed a significant increase in S-phase arrest in A549 cells (P < 0.0005). The CIS@PCL group exhibited a mild increase in S-phase arrest compared to the AgNPs@PCL group. Interestingly, both AgNPs@PCL and CIS@PCL induced S-phase arrest in A549 cells in this study. Therefore, the combined effects of CIS and AgNPs in CIS@AgNPs@PCL resulted in the most pronounced S-phase arrest, as illustrated in Figs. 4(d) and 4(i).

Double-strand DNA breaks (DSBs) represent the most severe DNA damage with γ-H2AX serving as a crucial biomarker for analyzing *in vivo* DSBs.^31^ In this study, immunofluorescence staining was provided to evaluate the relative expression of γ-H2AX. As shown in Fig. 4(e), the fluorescence in the AgNPs@PCL group was slightly higher than that in the PCL group, while the fluorescence expression in the CIS@PCL group was not lower than that in the AgNPs@PCL group. Due to the synergistic effects of AgNPs and CIS, the γ-H2AX fluorescence intensity was most pronounced in the CIS@AgNPs@PCL group. The above findings demonstrate that both AgNPs and CIS can promote DSBs in cellular DNA, thereby regulating tumor cell apoptosis. Moreover, CIS@AgNPs@PCL exhibited the most significant inhibition of tumor cells.

### D. *In vivo* LC recurrence model

**FIG. 5.**
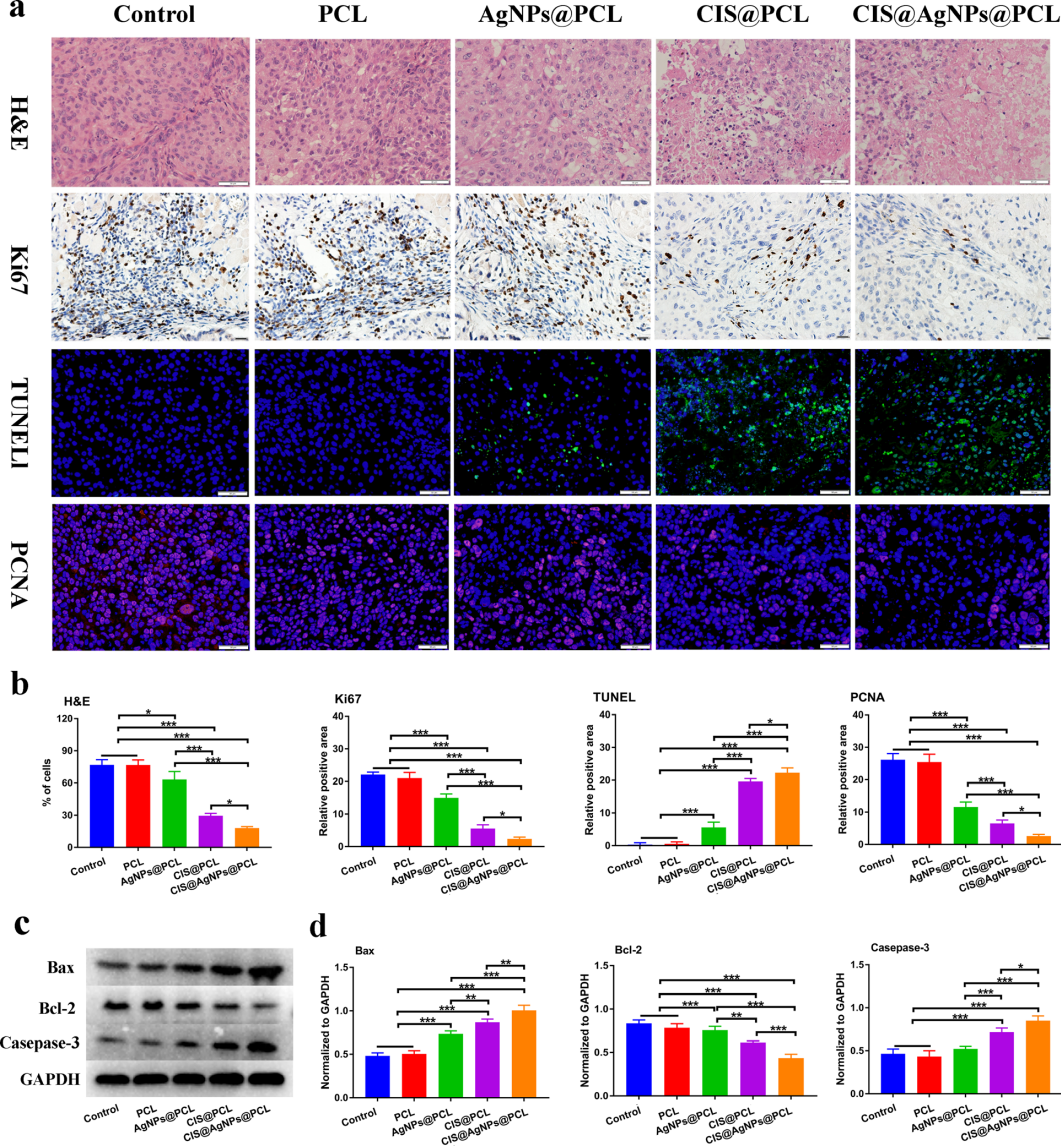
*In vivo* anticancer efficiency of electrospun drug-eluting stent membrane. (a) Representative photos of H&E staining, immunohistochemistry Ki67, TUNEL, and PCNA tumor proliferation after treatment in different groups. (b) Quantitative expression of H&E, Ki67, TUNEL, PCNA in tumors of five groups of experimental animals. (c) Relative positive area of a tumor section from Bax, Bcl-2, and Casepase-3. (d) Western blot analyses of expressed protein levels of Bax, Bcl-2, and Casepase-3 in tumor tissues. ^*^, P < 0.05; ^**^, P < 0.005; and ^***^, P < 0.0005.

MCAO caused by airway metastasis or compression of advanced LC is the main reason for asphyxiation death in patients. This study evaluated the efficacy of the different fiber membranes to prevent local tumor recurrence of LC in BALB/c nude mice. In brief, half of the tumor volume was resected when the mouse subcutaneous tumors reached 250–300 mm^3^. Subsequently, the membranes of PCL, AgNPs@PCL, CIS@PCL, and CIS@AgNPs@PCL were attached to the resected tumor surface and sutured to evaluate the antitumor efficacy of various fiber membranes [Fig. 5(a)].

A week post-surgery, the mice in all the experimental groups demonstrated positive physical health, characterized by a healthy complexion and absence of any eating abnormalities. However, after 15 days, the activity level of mice in the control and PCL groups showed a decline, and the skin surrounding the tumor site exhibited a purplish hue [Fig. 5(b)]. Additionally, fibrous membrane PCL has no definite change in tumor size, and the growth curve of tumor volume in this group is almost the same as that in the control group. The results revealed that the empty PCL fiber membrane and its degradation products did not affect the progression of tumor recurrence [Figs. 5(c) and 5(d)]. During the 18-day treatment cycle, the tumor volumes of the mice in the control, PCL, AgNPs@PCL, CIS@PCL, and CIS@AgNPs@PCL groups reached approximately 1124.75 ± 86.31, 1022.38 ± 48.05, 807.70 ± 102.22, 426.05 ± 42.35, and 239.50 ± 48.25 mm^3^, respectively. CIS@PCL exhibits stronger anti-tumor activity compared to AgNPs@PCL (P < 0.001) [Fig. 5(e)]. Importantly, the observed anti-cancer trend in the LC recurrence model aligns with the results of the cellular experiments. Specifically, due to the combined therapeutic effects of CIS and AgNPs, CIS@AgNPs@PCL demonstrates superior anti-tumor efficacy compared to CIS@PCL, indicating that the combination of CIS and AgNPs in electrospun nanofibers exerts the most potent anti-cancer effect. It significantly inhibits tumor proliferation *in vivo* and possesses the ability to improve the patency rate of MCAO.

### E. Pathological evaluation of tumors treated with electrospun fiber membranes

**FIG. 6.**
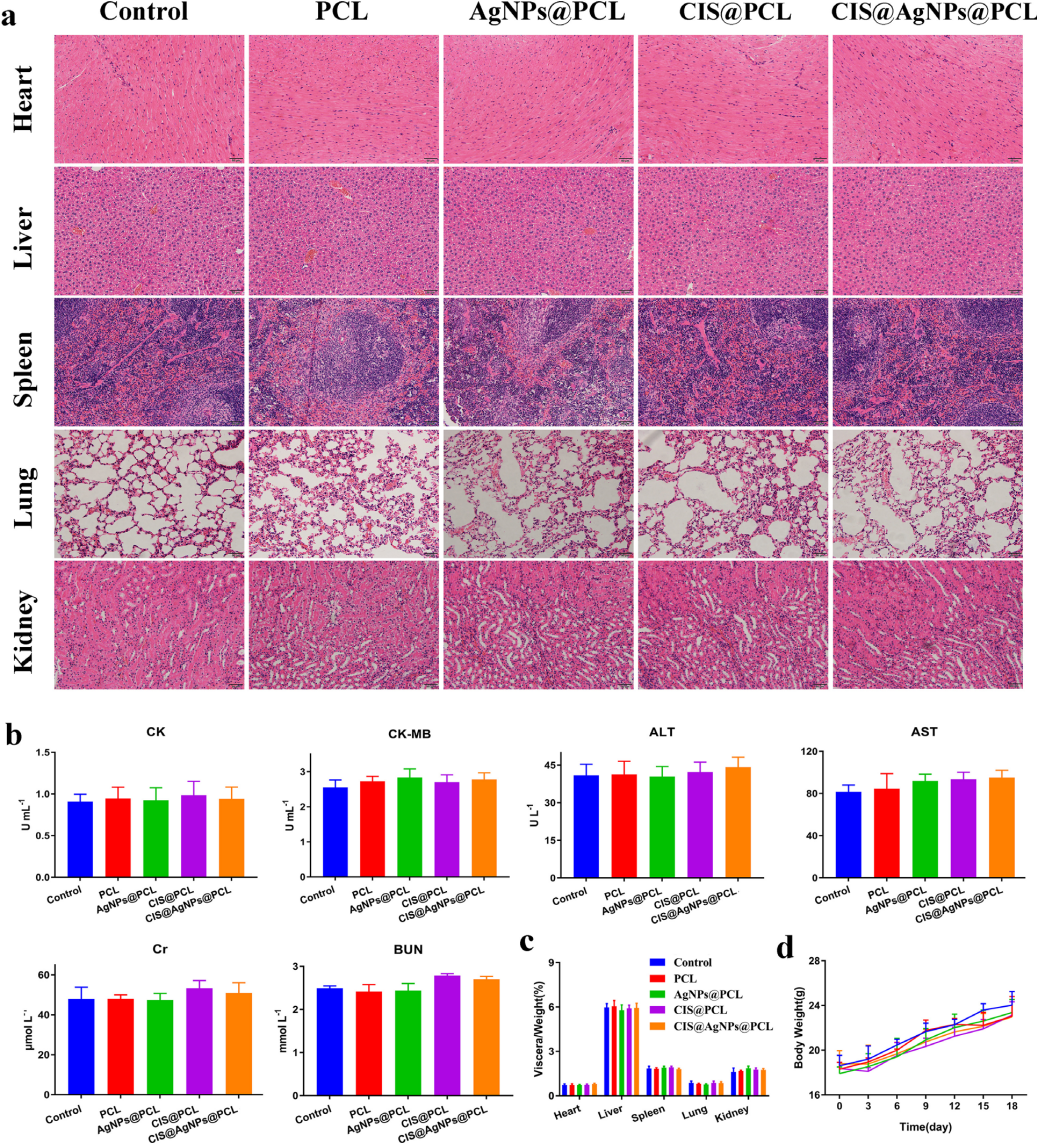
*In vivo* safety evaluation of different fiber membranes. (a) H&E staining of heart, liver, spleen, lung, and kidney tissues of nude mice 18 days after treatment in the control, PCL, AgNPs@PCL, CIS@PCL, and CIS@AgNPs@PCL group. (b) The examination of CK, CK-MB, ALT, AST, BUN, and Cr in serum when the animals were sacrificed. (c) Organ indices of A549 tumor-xenografted mice after treatment. (d)Trend of weight changes in five groups of mice.

We further examined the therapeutic efficacy of the CIS@AgNPs@PCL fiber membrane by typical morphological analysis [Fig. 6(a)]. The images of tumors stained with H&E from the control and PCL groups depicted nuclei morphology that was intact, suggesting a negligible degree of necrosis. In contrast, the AgNPs@PCL, CIS@PCL, and CIS@AgNPs@PCL exhibited varying levels of tissue necrosis with the most pronounced effect observed in the CIS@AgNPs@PCL group. Specifically, the percentages of necrotic tumor cells in the control, PCL, AgNPs@PCL, CIS@PCL, and CIS@AgNPs@PCL groups were 76.90% ± 4.10%, 76.85% ± 3.98%, 63.2% ± 6.5%, 29.50% ± 2.00%, and 17.95%±1.26%, respectively [Fig. 6(b)]. Ki67 index has been widely used as a marker of malignant tumor proliferation. Previous studies have shown that the low expression of Ki67 has a certain correlation with the clinical prognosis of non-small cell lung cancer, and the survival time may be prolonged.^32^ The CIS@AgNPs@PCL group showed a seriously lower expression of Ki67 compared to the other four groups, indicating that the cell proliferation was reduced and the fibrous membrane was better for tumor control. (P < 0.005). This observation was consistent with the PCNA immunofluorescence results. Additionally, the TUNEL staining assay revealed a higher number of apoptotic cells in tumors treated with CIS@AgNPs@PCL. Within the visual field, the proportion of apoptotic cells emitting green fluorescence increased gradually in all groups, but the increase was most significant in the CIS@AgNPs@PCL group. In brief, the proportion of apoptotic cells in the control, PCL, AgNPs@PCL, CIS@PCL, and CIS@AgNPs@PCL groups were 0.48% ± 0.43%, 0.73% ± 0.37%, 5.55% ± 1.40%, 19.57% ± 0.83%, and 22.27% ± 1.31%, respectively. These findings suggest that the synergistic anticancer properties of CIS and AgNPs contributed to the observed increase in apoptotic cells.

Research has indicated that the introduction of AgNPs triggers cancer cell apoptosis via various pathways, including the Bax/Bcl-2 and caspase pathways.^33^ In fact, the application of AgNPs in chemotherapy can enhance treatment efficacy and decrease drug resistance occurrences.^34,35^ The expression of Bax, Bcl-2, and caspase-3 proteins in tumor tissue was analyzed using Western blotting [Fig. 6(c)]. With the gradual release of AgNPs and CIS from the fiber membranes, the expression of Bax protein in the tumor tissue increased. Specifically, the Bax protein expression in the CIS@PCL group was higher than that in the control and PCL groups (P < 0.05), while the CIS@AgNPs@PCL group exhibited the highest level of Bax protein expression among all experimental groups (P < 0.05). Additionally, the protein expression trend of caspase-3 was consistent with the above results. Conversely, the fiber membranes loaded with AgNPs and CIS demonstrated an inhibitory trend in the expression of Bcl-2 in tumor tissue with CIS@AgNPs@PCL exerting the most significant regulation of Bcl-2 in the tumor tissue (P < 0.05) [Fig. 6(d)]. The study indicates that the fiber membranes loaded with AgNPs and CIS promote cell apoptosis by upregulating the Bax pathway and downregulating the Bcl-2 pathway. Moreover, CIS@AgNPs@PCL, which possesses synergistic anti-cancer capabilities, achieves better control of tumor proliferation and maintains airway patency.

### F. Safety of the electrospun fiber membranes *in vivo*

**FIG. 7.**
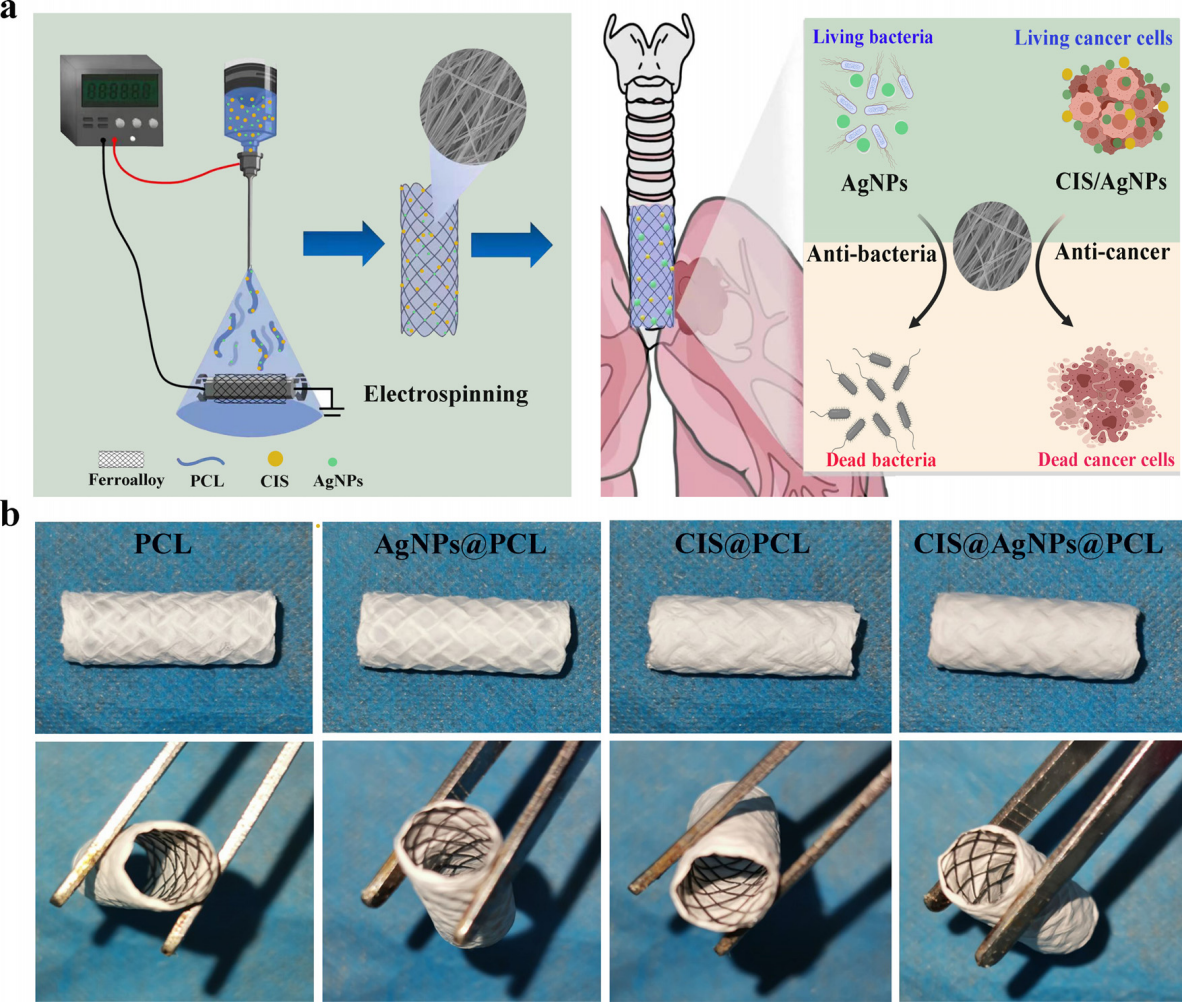
Fiber membrane-covered airway stent CIS@AgNPs@PCL was prepared by the electrospinning technique. (a) The construction process of the electrospun fiber membrane-covered airway stent. (b) Different orientations illustrate the specific shape of the four groups of fiber membrane-covered airway stents after preparation.

The clinical use of chemotherapeutic drugs and AgNPs is mainly limited by their serious complications and side effects. Therefore, the safety analysis of airway stent loaded with CIS and AgNPs is an important reference index for future clinical application. As shown in Fig. 7(a), H&E staining confirmed that the main organs of animals in each group, including heart, liver, spleen, lung, and kidney, had intact cells and tissue structures without substantial damage during the anti-cancer treatment. In addition, hematological and biochemical analysis showed that the liver and kidney function and cardiac function indices of animals in each group were within the normal range, and no potential toxicity was found [Fig. 7(b)].

Weight changes are also essential indicators for assessing the toxicity reactions of CIS and AgNPs after *in vivo* application. With the passage of time, the weights of all mice in the AgNPs@PCL, CIS@PCL, and CIS@AgNPs@PCL groups increased steadily, which is almost the same as the control group [Fig. 7(d)]. In addition, the evaluation of animal organ index by calculating the ratio of organ weight (mg) to body weight (g) is an effective parameter to reflect the toxicity of the tissue [Fig. 7(c)]. There were no significant differences between the experimental group and the control group in terms of heart, liver, spleen, lung, and kidney indices (P > 0.05). The above results confirmed that the membrane loaded with CIS and AgNPs does not cause serious systemic toxicity or liver/kidney dysfunction *in vivo*, suggesting that the newly constructed drug-loaded membrane airway stent CIS@AgNPs@PCL has high safety and potential clinical value in the future application.

## III. CONCLUSION

In our study, a series of fiber membrane-coated airway stents, including AgNPs@PCL, CIS@PCL, and CIS@AgNPs@PCL, were successfully fabricated by electrospinning to prevent MCAO and bacterial biofilm formation. This simple and effective design combines the excellent drug-loading function of the electrospun fiber membrane with the physical properties of the self-expanding metal stent, which can rapidly reconstitute the obstructed airway and release the drug locally. In brief, the CIS@AgNPs@PCL airway stent can (1) continuously locally release of CIS to the airway, which prevents the metastasis of lung cancer by up-regulating Bax, Casepase-3 and down-regulating the expression of Bcl-2 to maintain airway patency and prevent blockages, (2) inhibit pathogenic microorganisms in the respiratory tract and prevent the formation of bacterial biofilms, and (3) AgNPs induce disruptions in the DNA of cancerous cells, leading to enhanced efficacy of CIS-based chemotherapy. To summarize, this study presents a proof-of-concept approach demonstrating that airway stents can be modified to possess both anticancer and antimicrobial properties. This modification enables rapid relief of MCAO and sustained release of chemotherapy drugs to prevent intratracheal recurrence.

## IV. METHODS

### A. Materials

Poly(ε-caprolactone) (PCL; Mn = 100 000 g/mol) was purchased from Changchun SinoBiomaterials Co., Ltd. (Changchun, China). CIS and hexafluoro-isopropyl alcohol (HFIP) were purchased from Aladdin (Shanghai, China). AgNPs (d = 15–20 nm) were provided by Kunming Guiyan Pharmaceutical Co., Ltd. (Kunming, China). Nanjing Microtech Co., Ltd. (Nanjing, China) provided uncovered self-expanding metal stents (SEMS; 20 × 8 mm^2^). The Shanghai Cell Center of the Chinese Academy of Sciences provides NSCLC cells (A549). Bacterial species (*Pseudomonas aeruginosa*-ATCC 27853; *Staphylococcus aureus*-ATCC 25923) and fungi (*Candida albicans*-ATCC 10231) are provided by the China Type Culture Collection and Shanghai Institutes for Biological Sciences (SIBS). RPMI-1640 medium, fetal bovine serum (FBS), and penicillin/streptomycin were supplied by Sigma-Aldrich, Inc. (China). Thermo Fisher Scientific provided the Live/Dead BacLight bacterial viability kit, the Live/Dead cell viability kit, and the Annexin V-FITC apoptosis detection kit. Servicebio Biotechnology Co., Ltd. (Wuhan, China) provided the Ki67, TUNEL, Bax, caspase-3, Bcl-2, and PCNA antibodies.

### B. Sample preparation

**FIG. 8.**
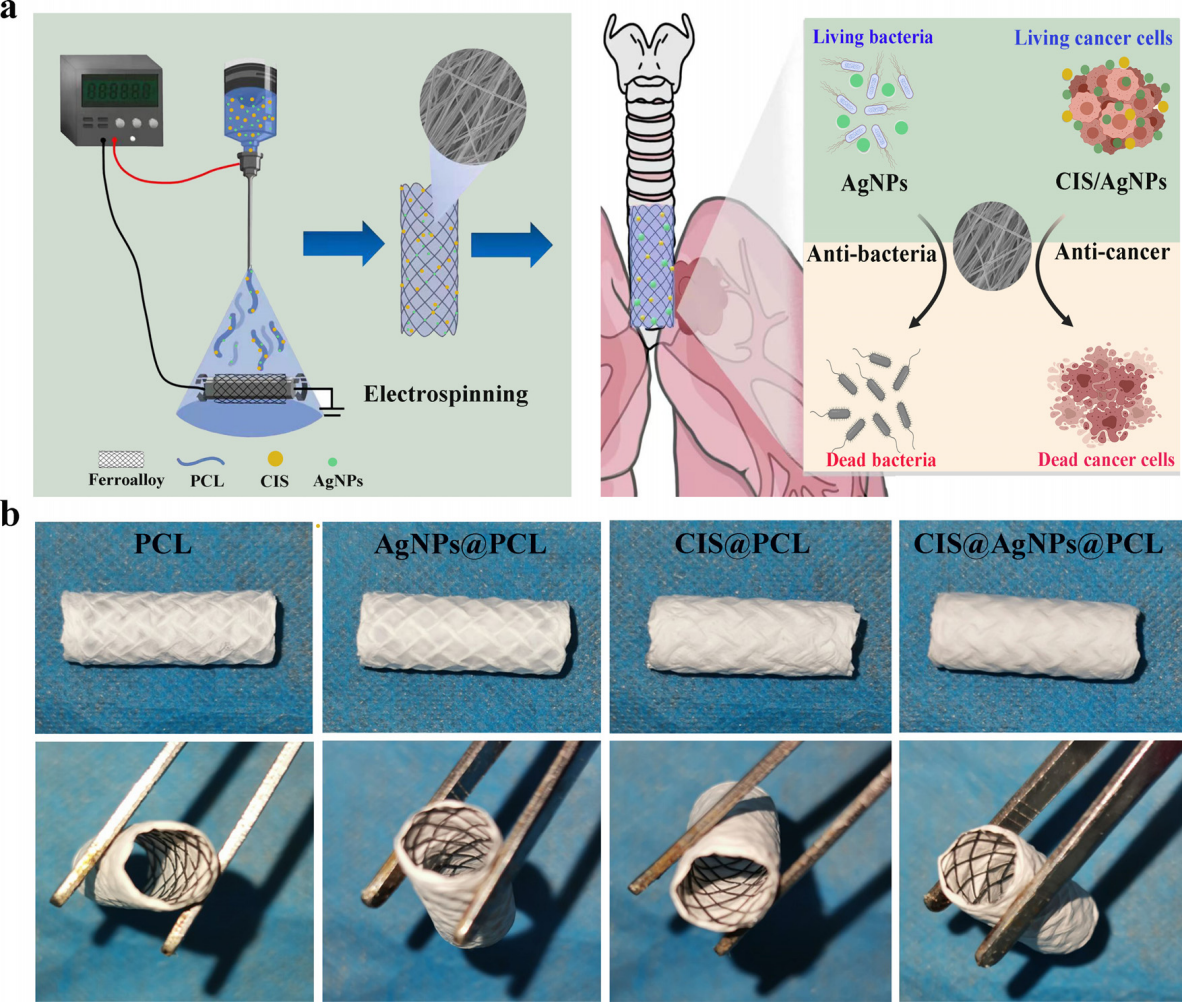
Fiber membrane-covered airway stent CIS@AgNPs@PCL was prepared by the electrospinning technique. (a) The construction process of the electrospun fiber membrane-covered airway stent. (b) Different orientations illustrate the specific shape of the four groups of fiber membrane-covered airway stents after preparation.

HFIP was used as the solvent for PCL, and the drug content in the fiber membrane was determined based on recommendations from previous reports.^36^ In short, first, 1.00 g of PCL was mixed with 8 ml HFIP and homogeneously dissolved by a magnetic stirrer to prepare a PCL solution carrier. Then, 0.04 g AgNPs was mixed with the PCL solution to successfully prepare an AgNPs@PCL solution with 4% AgNPs content. Similarly, as shown in Table I and Fig. 8(a), CIS@PCL and CIS@AgNPs@PCL with 4% CIS content were prepared. With a high-voltage DC power supply, the above four solutions were electrospun at a flow rate of 0.6 ml/h and a voltage of 16 kV, with a collector placed 18 cm away from the needle tip. SEMS was fixed to the collector to construct an electrospun fiber membrane airway stent. Successfully obtained drug-eluting fiber membrane airway stents were PCL, AgNPs@PCL, CIS@PCL, and CIS@AgNPs@PCL. All types of airway stents must be vacuum-dried for two days before use [Fig. 8(b)].

**TABLE I.** Synthetic component of fibrous membranes.

Sample	PCL (g)	AgNPs (g)	CIS (g)	HFIP (ml)
PCL	1.00	0	0	8
AgNPs@PCL	1.00	0.04	0	8
CIS@PCL	1.00	0	0.04	8
CIS@AgNPs@PCL	1.00	0.04	0.04	8

### C. Surface characterization

Chemical groups of the fibrous membranes were studied by Fourier transform infrared spectroscopy (ATR-FTIR, Nicolet 6700, USA) in the wavelength range of 4000–500 cm^−1^. The size distribution and morphological structure of AgNPs in the coated stent AgNPs@PCL and CIS@AgNPs@PCL fibers were observed and analyzed. In addition, the obtained PCL, AgNPs@PCL, CIS@PCL, and CIS@PCL@AgNPs fiber membrane samples were sputtered with gold for 120 s in a K-palladium vacuum coater, and then a scanning electron microscope (SEM, JSM-7401F, JEOL, Japan) equipped with an energy-dispersive spectroscopy (EDS) was used to identify the morphology characteristics and structure of the fiber membrane and analyze the element characteristics of the surface of the fiber membrane.

### D. *In vitro* drug release

Evaluation of the *in vitro* release behavior of AgNPs-containing fiber membranes was carried out as follows. First, films were made into samples weighing about 10 mg each and then completely immersed in 5 ml of constant temperature PBS. The solution was shaken at 50 rpm at a constant temperature. At predetermined time intervals (0–25 days), The amount of AgNPs released into the solution from the fiber membrane was determined by graphite furnace atomic absorption spectrometry (AAS9000, Suzhou Shipu Instrument Co, Ltd., China). Similarly, the amount of CIS released into the extraction bottle (PBS, pH7.4) from the fiber membrane CIS@PCL and CIS@AgNPs@PCL was analyzed by UV-visible spectroscopy (ND-ONE-W-A30221, Thermofisher, United States) at 220 nm.

### E. Agar diffusion assay

Each sample was prepared as a circle with a diameter of 0.5 cm, and the front and back sides were irradiated with ultraviolet lamps for 30 min for disinfection. The revived *S. aureus*, *P. aeruginosa*, and *C. albicans* were used to prepare microbial suspensions (1 × 10^6^ CFU/ml where CFU is a colony forming unit; 100 *μ*l), which were spread on LB solid medium. Each sample was placed on a plate and incubated under aerobic conditions at 37 °C for 24 h, and the diameter of the inhibition zone was recorded.

### F. Biofilm formation experiment

Make a sample of each group of fiber membranes with a size of 1 × 1 cm^2^ and place it in a 6-well plate, then add 60 *μ*l of microbial suspension (4.8 × 10^9^ CFU/ml) and LB broth to 3 ml and continue to incubate in the incubator overnight. The medium was changed after 24 h to allow the microorganisms to adhere to the material surface. The samples were further incubated, and mature biofilms had formed within 72 h. After incubation, the impurities on the sample surface were washed with 0.85% NaCl solution three times to remove nonadhered microorganisms. Biofilms were stained with the reagents in the Live/Dead BacLight bacterial viability kit, which included the dyes DMAO and EthD-III, and then the samples were incubated in the dark (15 min). Finally, the biofilms were imaged by confocal laser scanning microscopy (CLSM). The total microbial load on the membrane surface was evaluated by COMSTAT2 software.

### G. *In vitro* cell experiments

To examine the *in vitro* antitumor properties of PCL, AgNPs@PCL, CIS@PCL, and CIS@AgNPs@PCL fiber membranes, NSCLC cells (A549) were selected as model cells.

### H. CCK-8 assay

Evaluate the effect of four-group fibrous membranes on inhibiting the proliferation of A549 lung cancer cells by Cell Counting Kit-8 (CCK-8). In short, incubate PCL, AgNPs@PCL, CIS@PCL, and CIS@AgNPs@PCL in the culture medium and collect the release solution at 24, 48, and 72 h, respectively. Then transfer the cells to 96-well plates at a concentration of 200 *μ*l per well (3.3 × 10^4^/ml) until the cells are completely attached to the wall, and continue to incubate with the cells for 24 h with the release solution obtained in the previous stage. Afterward, wash the cells twice with PBS, add 10 *μ*l CCK-8 reagent, and incubate in a darkroom at 37° for 2 h. The 96-well plates were measured at 450 nm for the optical density (OD) of each experimental group with a microplate reader (Synergy 4, BioTek, USA) after coloration.

### I. Live/dead cell staining assay

The study of the live/dead situation of four groups of fibrous membranes on A549 cells was based on the experimental steps of the manufacturer. In brief, A549 cells were cultured in a 37 °C incubator at a cell density of 4.2 × 10^4^ cells per well overnight until they completely adhered to the wall, then replaced with the culture mediums of PCL, AgNPs@PCL, CIS@PCL, and CIS@AgNPs@PCL to incubate for 24 and 48 h. Afterward, the releasing solution was removed, and the cells were thoroughly rinsed with PBS for three times. A cell dye was prepared by uniformly mixing Calcein-AM and EthD-I at room temperature. 200 μl of the dye was added to each well and incubated at room temperature in the dark for 25 min, then the live/dead status of the cells was observed and captured in a fluorescence microscope (Carl Zeiss, Germany).

### J. Apoptosis assay

The release solution was collected after culturing the four groups of fibrous membranes with the cell culture medium for 24 and 48 h. A549 cells were placed overnight in a 37 °C incubator at 2.3 × 10^5^ cells per well until they were completely adhered to the wall, then replaced with the culture mediums obtained earlier to continue culturing with the cells for 1 day. Then, the cells were collected at a concentration of 1.2 × 10^6^ in centrifugation tubes and washed with a 4° PBS solution for three times. According to the manufacturer's scheme, Annexin V-FITC Apoptosis Detection Kit was used.

### K. Cell cycle assay

A549 cells were incubated overnight in a six-well plate with a density of 2.3 × 10^5^ per well for 24 h in the incubator. After changing the medium and adding the PCL, AgNPs@PCL, CIS@PCL, and CIS@AgNPs@PCL release solution, the cells were incubated for one day before being collected and adjusted to a cell concentration of 1.1 × 10^6^/ml. Then the cells were fixed overnight with 70% ethanol in a 4 °C refrigerator, washed twice with PBS before use, and the samples were detected by flow cytometry (BD Accuri® C6 Plus, USA) according to the requirements of the cell cycle kit.

### L. γ-H2AX expression

A549 cells were grown overnight in the incubator at a density of 4.2 × 10^4^ per well. The medium was changed, the PCL, AgNPs@PCL, CIS@PCL, CIS@AgNPs@PCL release solution was added, and the cells were incubated for 24 h. After that, the cells were fixed with 4% polyformaldehyde for 15 min. The immunofluorescence (IF) staining of γ-H2AX was completed according to established protocols.

### M. Lung cancer recurrence model

The animal experiments in this study were approved by the Animal Care Committee of the First Affiliated Hospital of Zhengzhou University. First, 25 female BALB/c nude mice were prepared. Additionally, A549 cells cultured to the logarithmic phase of growth were harvested. The backs of the nude mice were disinfected and subcutaneously injected with 100 *μ*l/7.7 × 10^6^ cells, and partial tumor resection was performed after the subcutaneous tumors reached a volume of approximately 250–300 mm^3^ within 2–3 weeks. Specifically, animals were anesthetized by intraperitoneal injection of ketamine solution, a small incision was made in the dorsal skin to expose the tumor, and half of the original tumor volume was excised. The nude mice were randomly divided into five different groups (5 mice/group): control, PCL, AgNPs@PCL, CIS@PCL, and CIS@AgNPs@PCL. The treatment plan was implemented on day 0, and photographs of representative mice and isolated tumors from each group were taken at the end of treatment on day 18. All experimental animals were subcutaneously injected with 5 mg/kg meloxicam within three days after the surgical operation to relieve pain. Of note, animal body weight and recurrent tumor volume were recorded during treatment. In addition, tumors were collected after all mice were euthanized after 18 days of treatment. The antitumor efficacy of PCL, AgNPs@PCL, CIS@PCL, and CIS@AgNPs@PCL fiber membranes was assessed by determining the tumor volume (V), which was calculated using the following equation:^37^

$$V(mm^{3})=\frac{L\times S^{2}}{2},$$

where L (mm) is the length of the long axis of the tumor and S(mm) is the length of the short axis of the tumor. Additionally, record the weight of major organs in the animal. Evaluate the animal's organ index using the following equation:

$$\mathrm{Organ}\;\text{index}\left(\%\right)=\frac{W\;\mathrm{Organ}}{W\;\text{body}}\times 100\%.$$

### N. Western blot analyses

The frozen tumor tissues were homogenized, and the protein content was determined using the BCA protein assay kit (A53225, Thermo Fisher Scientific, USA). Five samples were separated by SDS-PAGE and transferred to the PVDF membrane. After four times of TBST washing, sealing was completed by 5% skim milk. Then it was incubated overnight with primary antibodies against Bax, Bcl-2, and caspase-3. After two washes with PBS, the membrane was further incubated with secondary antibodies. Finally, it was washed and exposed with an ECL kit (Millipore, USA). ImageJ software was used for quantitative analysis of bands to detect the expression of apoptotic proteins in the tumors.

### O. Histopathological, immunohistochemical, and biochemical analyses

After the animals were sacrificed, the hearts, livers, spleens, lungs, kidneys, and tumor tissues of the mice were collected, fixed in 4% (w/v) paraformaldehyde for 24 h, and then embedded in paraffin. The embedded tissue sections were deparaffinized and stained with H&E, which were observed with a fluorescence microscope. The data of each group were then analyzed using ImageJ software. Moreover, the expression of Ki67, TUNEL, and PCNA proteins in tumor tissues was evaluated by immunofluorescence and immunohistochemistry, and the data of each group were analyzed using fluorescence microscopy (Olympus, Japan).

### P. Safety assessment

To further confirm the safety of the fiber membranes *in vivo*, a commercially available ELISA kit (Shanghai Lichen Biotechnology Co., Ltd., China) was used to detect the corresponding functional enzymes in the blood of the mice. In brief, according to the standard protocol provided by the supplier, ELISA kits were used to detect the levels of liver-associated alanine aminotransferase (ALT), creatine kinase (CK), creatine kinase-MB (CK-MB), aspartate aminotransferase (AST), kidney-related blood urea nitrogen (BUN), and creatinine (Cr).

### Q. Statistical analysis

Statistical analysis was conducted using SPSS (IBM Corp, New York, USA) or GraphPad Prism 8.0 (GraphPad Software, Inc., La Jolla, USA). All experimental data were expressed as the mean ± standard deviation. Two-way ANOVA or Student's t test was used for data examination, and statistically significant values were marked with ^*^P < 0.05, ^**^P < 0.005, and ^***^ P < 0.0005.

## Data Availability

The data that support the findings of this study are available from the corresponding authors upon reasonable request.
